# A Robot-Assisted Therapy to Increase Muscle Strength in Hemiplegic Gait Rehabilitation

**DOI:** 10.3389/fnbot.2022.837494

**Published:** 2022-04-29

**Authors:** Javier Gil-Castillo, Patricio Barria, Rolando Aguilar Cárdenas, Karim Baleta Abarza, Asterio Andrade Gallardo, Angel Biskupovic Mancilla, José M. Azorín, Juan C. Moreno

**Affiliations:** ^1^Neural Rehabilitation Group, Cajal Institute, Spanish National Research Council (CSIC), Madrid, Spain; ^2^Research and Development Unit, Rehabilitation Center Club de Leones Cruz del Sur, Punta Arenas, Chile; ^3^Electrical Engineering Department, Universidad de Magallanes, Punta Arenas, Chile; ^4^Systems Engineering and Automation Department, Universidad Miguel Hernández de Elche, Elche, Spain; ^5^Universidad Católica de Chile, Santiago, Chile

**Keywords:** stroke, hemiparesis, cerebrovascular disorders, brain diseases, robot therapy, gait rehabilitation

## Abstract

This study examines the feasibility of using a robot-assisted therapy methodology based on the Bobath concept to perform exercises applied in conventional therapy for gait rehabilitation in stroke patients. The aim of the therapy is to improve postural control and movement through exercises based on repetitive active-assisted joint mobilization, which is expected to produce strength changes in the lower limbs. As therapy progresses, robotic assistance is gradually reduced and the patient's burden increases with the goal of achieving a certain degree of independence. The relationship between force and range of motion led to the analysis of both parameters of interest. The study included 23 volunteers who performed 24 sessions, 2 sessions per week for 12 weeks, each lasting about 1 h. The results showed a significant increase in hip abduction and knee flexion strength on both sides, although there was a general trend of increased strength in all joints. However, the range of motion at the hip and ankle joints was reduced. The usefulness of this platform for transferring exercises from conventional to robot-assisted therapies was demonstrated, as well as the benefits that can be obtained in muscle strength training. However, it is suggested to complement the applied therapy with exercises for the maintenance and improvement of the range of motion.

## Introduction

Stroke is a leading cause of death and disability worldwide, with an incidence of nearly 14 million new cases each year (Johnson et al., 2019). The survivors have severe motor impairment, such as hemiparesis, which affects 65% of victims (Wist et al., 2016). Two of the main disorders after stroke are reduced muscle strength and spasticity (Thibaut et al., 2013). On the one hand, muscle weakness contributes to limited mobility and is related to poor performance in functional activities. In addition, the neural mechanisms that control muscle strength involve the recruitment of motor units and are altered and disrupted after a stroke. This recruitment depends on the task and the rate of motor units already active (Chisari et al., 2015). Therefore, muscle strength may be an appropriate target for therapeutic interventions (Jeon and Hwang, 2018; Tieland et al., 2018). In fact, it has been observed that the strength of the hip flexor and knee extensors of the hemiplegic limb are the most important factors determining appropriate or rapid gait speed (Wist et al., 2016). On the other hand, spasticity is involved in the development of limitations in joint Passive Range-Of-Motion (PROM) after stroke, which is another common musculoskeletal problem (De Bruin et al., 2013). Muscle weakness and spasticity lead to dysfunctions in gait biomechanics, resulting in inefficient and abnormal gait patterns. These impairments cause many difficulties in carrying out daily activities and mobility, reducing people's quality of life. Therefore, people with this condition have limitations such as low gait speed, gait pattern dysfunctions and an increased risk of falls (Li et al., 2018). Furthermore, gait pattern dysfunctions lead to high metabolic costs mainly related to compensatory movements in non-affected joints (e.g., trunk flexion, hip circumduction, or excessive flexo-extension in the hip and knee joints) (Gomez-Vargas et al., 2021).

Stroke rehabilitation is primarily focused on gait recovery. Thus, conventional therapy combines ground gait training with other exercises needed for gait rehabilitation including stretching, strengthening, endurance, balance, coordination, and range-of-motion activities (Bae et al., 2014; Guzik et al., 2018). Gait rehabilitation is necessary because training without therapeutic intervention can lead to an asymmetric pattern with problems in postural control and dysfunctions in muscle activation during gait (Bae et al., 2014). However, it requires considerable time and physical effort from the therapists. Thus, it also limits the number of patients a physiotherapist can treat (Díaz et al., 2011; Bryce et al., 2015). The disadvantages of conventional therapy have led to the design and development of other methods that facilitate treatment, such as functional electrical stimulation, robotic devices, electromechanical devices, and brain-computer interfaces, among others (Belda-Lois et al., 2011).

In particular, robotic assistance may offer certain advantages over conventional therapy. These include a standardized training environment, adaptive support, and increased training intensity and dosage (Gassert and Dietz, 2018). Robotic assistance also allows therapists to provide the same traditional therapy but reducing time and physical effort, and increasing the number of patients and therapies provided (Díaz et al., 2011). Furthermore, robotic assistance helps in control of speed, range of motion and coordination patterns, providing weight bearing, and enabling more reliable standardized therapeutic procedures (Bryce et al., 2015). In this sense, robotic assistance also facilitates the work of clinicians who are responsible for setting up the system and supervising therapy. Robotic therapy can also be used to train the patient to acquire a functional gait pattern that avoids pathological movement compensations (Díaz et al., 2011).

However, the clinical application and impact of these technologies are still limited. One limiting factor is that robotic devices are often heavy and bulky and must be used under supervision and with technical aids (Rodríguez-Fernández et al., 2021). In addition, knowledge exchange between bioengineering and clinical areas has been limited due to the technological focus of many research groups. As a consequence, there are few clinical trials demonstrating the efficacy of robot-assisted therapy and they are often limited to short studies with few participants. This, coupled with the fact that wearable exoskeletons for gait rehabilitation are in the early stages of development, means that most systems have not been clinically evaluated (Lajeunesse et al., 2015; Contreras-Vidal et al., 2016; Dijkers et al., 2016; Louie and Eng, 2016; Rodríguez-Fernández et al., 2021). While it is clear that a rehabilitation approach based on neurophysiological and clinical knowledge is necessary to achieve a positive effect, the lack of consensus for the optimal therapeutic program hinders the evaluation of these technologies in the clinical environment. This is due to a lack of understanding of the mechanisms of recovery and results in different outcomes in the literature. While there are clinical trials reporting superiority of gait rehabilitation using robotic therapy alone or in combination with conventional therapy, others report some non-significant improvement or that conventional therapy was superior. Therefore, to achieve a positive effect, a rehabilitation approach based on neurophysiological and clinical knowledge is necessary (Cao et al., 2014; Taveggia et al., 2016; Nolan et al., 2020; Infarinato et al., 2021).

Current evidence suggests that the intensity and dose of physical therapy play a key role in recovery. Furthermore, active physical and cognitive involvement of patients during therapy is crucial, which has promoted the use of adaptive assistance, automatic adaptation of task difficulty and motivational feedback (Gassert and Dietz, 2018). Moreover, locomotor training has also been shown to be more effective when performed in a real environment, which promotes the use of wearable exoskeletons. They are faster and more agile by increasing the strength capacity of muscles and also by providing a robust mechanical energy dissipation to prevent injury during high impact activities (Gassert and Dietz, 2018; Sawicki et al., 2020). However, the challenge of achieving low output impedance together with the provision of assistance is a critical point that limits the degrees of freedom of the system, increasing the complexity with respect to fixed systems. Despite the complexity of the technology and the milestones that remain to be reached, robotic assistance is a promising tool to complement conventional therapy in the clinic, offering great potential for continuous therapy and home care through simpler devices (Gassert and Dietz, 2018).

The goal of this type of assistance is to minimize unwanted abnormal activation patterns by minimizing the difference between normal and paretic limb movement, while increasing the repeatability and intensity of the training (Hobbs and Artemiadis, 2020). In fact, robotic therapy has shown to be effective in improving balance, strength, gait performance and motor skills required by high-severity stroke patients to perform activities of daily living (Sun et al., 2013; Cho et al., 2015; Chung, 2017; Kim et al., 2019). Its advantages and the results obtained have led robotic therapy to become a very popular gait rehabilitation technique worldwide and a standard treatment in stroke rehabilitation (Kasal and Takeda, 2016).

From a clinical point of view, the treatment method is also an important point and one of the main therapeutic approaches in stroke patients is the Bobath concept (Belda-Lois et al., 2011). The Bobath concept is considered the most widely used neurorehabilitation approach worldwide, as it focuses on motor recovery rather than compensation. It is an individualized, inclusive, problem-solving and life-solving concept that is based on the systems approach to motor control. It has an emphasis on motor recovery and movement analysis integrating task performance, postural control and contribution of sensory inputs (Vaughan-Graham et al., 2019). This method considers a relationship between movement and spasticity, considering muscle weakness due to the opposition of spastic antagonists. The Bobath method addresses increased muscle tone (spasticity) through passive mobilization associated with proprioceptive and tactile stimuli (Yadav et al., 2018). It also focuses on addressing task performance to identify the level of impairment or the level of participation, depending on the individual and the context in which the therapy is applied. Compensation can be minimized to obtain optimized function (Vaughan-Graham et al., 2019).

Therefore, the Bobath concept uses techniques aimed at normalizing muscle and postural tone to correct these abnormal patterns and facilitate walking. It focuses on restoring normal movements through re-education. This method uses techniques based on facilitation, therapeutic manipulation and activation of key control points, aimed at improving patients' motor control while using the different stages of normal motor development as a guide (Balzer, 2018). The Bobath concept is as effective method as other therapies and can be considered more effective than a standard rehabilitation process for the treatment of the lower limbs (Mikołajewska, 2017; Gray and Ford, 2018). In addition, it significantly improves basic mobility skills and balance. The advantage of including this method lies in the multi-repetitive, task-oriented approach and, due to these components, it has a direct impact on the level of disability (Mikołajewska, 2017; Gray and Ford, 2018; Huang et al., 2021). Furthermore, recent studies of the Bobath method have demonstrated improvements in cadence, gait speed and stride length, making it a more effective form of gait post-stroke rehabilitation compared to traditional rehabilitation (Mikołajewska, 2017).

Rehabilitation studies with cyclic gait in Lokomat have found improvements in walking ability with a significant increase in muscle activation rate not accompanied by an increase in strength (Chisari et al., 2015). This could suggest a training effect on motor neuron activation rate which therefore contributes to improved motor control (Chisari et al., 2015). However, this has not been evaluated in clinical trials with lower limb exoskeletons during selective voluntary motor control exercises (Kusumoto et al., 2016). Therefore, the aim of this study is to determine the changes in voluntary muscle strength and join range of movement after robot-assisted therapy based on Bobath treatment exercises. Thus, its use will be evaluated in combination with exercises based on the Bobath techniques for gait rehabilitation in stroke patients. These exercises were based on repetition of active-assisted joint mobilization tasks, which were expected to produce changes in muscle strength of the lower extremities.

## Materials and Methods

The present clinical study was led by the Rehabilitation Center Club de Leones Cruz del Sur and was registered in the ClinicalTrials.gov database (NCT number: NCT04228224, registration date 01/14/2020), a resource provided by the U.S. National Library of Medicine. The protocol (Code: CRCS_UID_210619) was approved by the Institutional Review Board of the Rehabilitation Center Club de Leones Cruz del Sur.

### Patients

A total of 23 patients (7 female and 16 male) with a mean age of 53.9 ± 9.7 years, mean weight of 77.7 ± 14.2 kg, and mean height of 163.8 ± 7.7 cm participated in this clinical study. All the participants were recruited from the Outpatient Rehabilitation Program of the Rehabilitation Center Club de Leones Cruz del Sur.

#### Eligibility Criteria

All the patients included in the present study had unilateral lower extremity paresis resulting from a stroke occurring at least 6 months prior to the study. Furthermore, patients had full passive range of motion in the lower extremities or at least reached a neutral joint position; they were also able to stand freely and walk with or without assistance for at least 20 m in <2 min. Exclusion criteria were peripheral nervous system pathology, epilepsy, weight over 100 kg, difficulty following the study instructions, pregnancy, use of implanted devices, and unstable lower extremity joints or fixed contracture. The co-researchers obtained written informed consent from all participants. All enrolled participants were informed of their responsibility to attend all scheduled sessions.

### System

**Figure 1:**
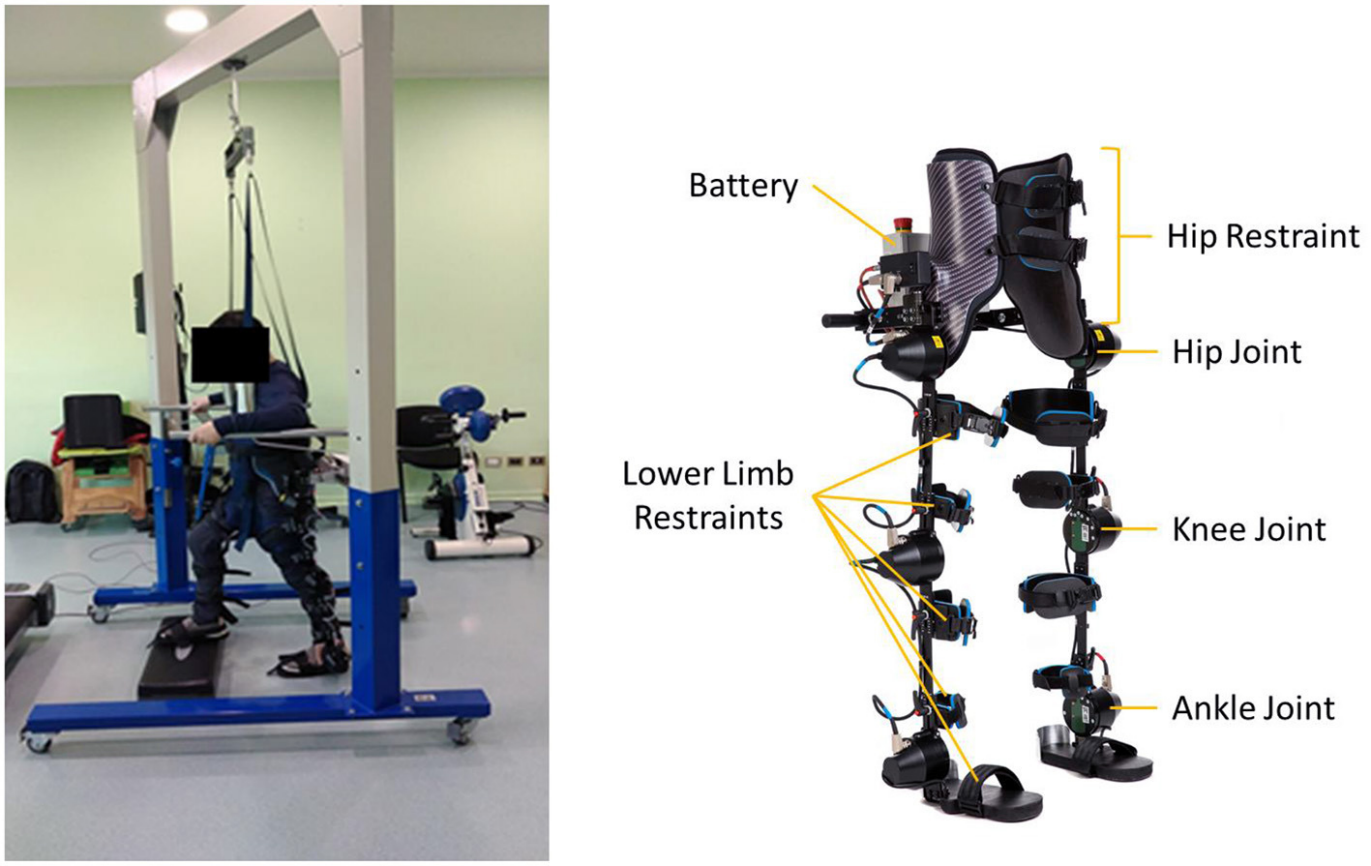
Exoskeleton platform—H3.

A rehabilitation platform was developed consisting of a powered lower limb exoskeleton (H3 Exoskeleton, TECHNAID, S.L., Spain), a control software and a weight-bearing system (Barría et al., 2022). This platform was the one used to apply the therapy. The H3 exoskeleton (Figure 1) consists of 6 motors and assists the movement of the lower limbs in the sagittal plane through electric motors aligned with the patient's joints axes. In addition, it detects the movements executed by the patient in the sagittal plane through position (magnetic encoders) and force (strain gauge) sensors located in the joint axis and force sensors located on the sole of the exoskeleton's feet. All sensor data was stored in a database for future studies. The architecture of the exoskeleton facilitates the control of the range of motion with robotic assistance in each motor separately and, as a consequence, in each joint independently. This assistance can be gradually adjusted according to the patient's remaining movement and lower limb muscle function. As a consequence of the joint-specific assistance synchronized with the voluntary movement of the patients, an individualized and adjustable locomotion training was designed for the bilateral hip, knee, and ankle flexors and extensors.

To control the assistance and performance of each of the exercises, a new software implemented in LabVIEW (LabVIEW, National Instruments, USA) was designed to control the position of each joint of the exoskeleton independently, adjust the assistance and record data from each rehabilitation session. The control diagram can be found in the supplementary material provided (Supplementary Figure S1). This software allows the configuration of the assistance variables provided by the H3 exoskeleton for position, torque and admittance. It also uses sagittal kinematic profiles pre-recorded by a photogrammetry system (VICON) and organized in the form of cycles. These cycles are repeated throughout the session in which the physiotherapist manages the rest times between each exercise, the speed of the movement and the number of repetitions. The exercises can be customized according to the patient's needs and capabilities by adjusting the minimum and maximum angles, the degrees of mobility and the percentage of assistance per joint. In addition, the program's graphical interface allows the therapist to view the programed curve, the curve executed by the patient in real time and the real-time updated average of the cycles performed during the session, making it easier for the therapist to recognize the movements that are most difficult for the patient and the range limitations of each exercise (Supplementary Figure S2).

The assistance level can be set in percentage values between 50 and 100% (Supplementary Figure S2), the latter being equivalent to 35 Nm. The exoskeleton provides continuous assistance from the start to the end of the cycle of each exercise and there are no real-time adjustments or modulation. It is possible to release the motors (zero torque), allowing free movement of the joints of both limbs without any assistance. In fact, as a safety measure, the torque of all joints was set to zero if the difference between the programed angle and that executed by the patient in real time was ≥10° at any joint. In therapy, torque is provided throughout the exercise cycle according to the initial assist percentage setting established for the session. The onset of exercise and assistance is anticipated to the patient by an alert sound, emitted by the LabVIEW interface to allow the patient to voluntarily accompany the movement according to his or her capabilities.

In short, the new platform allows to control the movement of each joint, facilitating normal movement and avoiding the use of compensatory movement strategies that patients use due to muscle weakness. Additionally, the software allows the modification of variables such as speed, repetitions and rest time between each exercise, facilitating the application of adjustments in each session to personalize the therapy according to the patient's progress.

### Experimental Protocol

Participants were assessed at baseline and after the robotic therapy through standardized clinical tests to measure the strength and PROM of each joint. Lower limbs PROM assessment was performed using a six-piece goniometer set (Jamar TECH, USA). Strength assessment was carried out using a digital handheld dynamometer (Commander Echo Wireless Muscle Testing, JTECH MEDICAL INDUSTRIES INC, USA) in which maximum and average muscle strength of hip flexion, hip extension, hip abduction, knee extension, knee flexion, dorsiflexion and plantar flexion of the ankle were assessed. The assessment was performed using the evaluation protocols available in the literature to reach positions with higher reliability to test isometric strength (Mentiplay et al., 2015).

After the assessment, patients participated in robot-assisted therapy with a lower extremity powered exoskeleton, which involved 24 sessions, 2 sessions per week for 12 weeks, each session lasting ~1 h. The sessions were scheduled with the aim of achieving 70% of assistance per patient. Even so, in the first session participants had full attendance (100%) and then gradually decreased their assistance by 5% every 4 sessions based on clinical judgment. Each session was subdivided into preparatory activities, robotic therapy and post-therapy activities. Preparatory activities included securing the patient by installing a harness connected to a non-movable weight-bearing system whose sole purpose is to secure the patient and prevent falls, but without supporting a fixed percentage of weight; installing and adjusting the exoskeleton; connecting the software; selecting the exercise to be performed, and setting the percentage of assistance per joint. In addition, vital parameters as blood pressure and heart rate were measured before and after each session with the SureSingVS2+ multiparametric device (Philips, Andover, MA, USA) to ensure that the patient performed the training in perfect condition and that no adverse effects occurred after the session.

The therapy design included 5 gait training exercises (Supplementary Figures S3–S6) based on kinematic data recorded from healthy subjects through an optoelectronic motion capture system (Vicon Oxford Metrics, UK.). The therapy consisted of 3 series of 15 repetitions; with a pause between each series of ~1–3 min; and a rest of ~2–5 min between exercises, depending on patient fatigue. The selected rehabilitation exercises corresponded to specific activities of the Bobath concept, which focuses on training the different phases of gait with therapist assistance. The training included gait cycle exercises focused on the stance and swing phases (Huang et al., 2021). The first exercise (step without load) consisted of lifting one leg and placing the foot in a forward step without weight discharge. The second exercise (step with load) consisted of performing the same movement as the previous exercise, but this time with weight offloading. The third exercise (pre-gait exercise) consisted of performing from a standing position with parallel feet, one step forward and one step backward, imitating a normal gait sequence. The fourth exercise (stand-to-sit transfer) consisted of performing the action of sitting on a chair from a standing position. The fifth and final exercise (sit-to-stand transfer) consisted of performing the action of rising from a seated position in a chair to a standing position. Patient safety was ensured throughout the training session. If the patient felt unwell, the exercise was stopped, the patient was sat down and vital parameters were measured again.

Once the training session was over, the system was switched off and the equipment was uninstalled. A skin inspection was carried out in the areas where pressure was applied with the exoskeleton and the patient was consulted about his or her comfort and experience during the session. In addition, at the end of each session the therapist monitored established criteria for discontinuing the study: (1) Participant requests to leave the study; (2) Follow-up assessment reveals evidence of unexpected contraindication to the intervention, such as skin problems or pain. In addition, participants' adherence to the program was monitored and encouraged throughout the trial. In addition, all patients were informed about permitted and prohibited concomitant interventions during the trial.

After 12 weeks of training, assessment tests were repeated for comparison with the baseline condition. In addition, post-trial care of the study included: (1) All patients enrolled in the study were scheduled for clinical follow-up; (2) Implementation of a telephone line for patients in case of post-trial complications.

#### Confidentiality

Only institution employees, co-researchers, and ethics committees get access to the participants' records. Participants' identities are concealed in any research related publications.

### Statistical Analysis

The data collected with the pre- and post-therapy assessments were analyzed with SPSS Statistics 26 software. The results obtained in the dynamometry tests were normalized by the weight of each subject in order to carry out the statistical study, so the units of the strength results presented in this study are kgf·kg^−1^. The results presented in relation to the PROM are in degrees. Descriptive statistics, such as mean (M) and standard deviation (SD), were calculated. An analysis of the classical assumptions was performed, i.e., normality tests (Shapiro Wilks test). The results obtained from these tests conditioned the use of a parametric (Student's *t*-test) or non-parametric (Wilcoxon test) test for related samples at 5% confidence. In addition, a statistical power study was performed with GPower v3.1 software to confirm the reliability of the results obtained with the proposed statistical analysis. For this purpose, it was established that the statistical power should be equal to or >80%. Due to the multiple comparisons made to identify significant differences in the variables measured for strength and PROM, the familywise error rate increases. That is, the probability of making a type I error increases. To control this effect, a Holm-Bonferroni adjustment was applied to the strength and PROM family variables separately. This allowed us to identify the significant differences for each of the groups of variables studied.

Finally, to assess the possible relationships between the significant changes detected, the percentage of change that occurred between the pre- and post-variables was calculated. For this purpose, the following equation was applied:


$$\%\;Variation_{PRE-POST}=\frac{\left(Post\;Value-Pre\;Value\right)}{Pre\;Value}100$$

Subsequently, the relationships between these newly calculated variables were evaluated for two different scenarios. Variations of the variables on the paretic side and on the non-paretic side were analyzed separately. In a first scenario, the relationship was studied for the variations of the maximum strength measures. They were only examined for maximum strength, as maximum strength and average strength were considered to be linearly dependent. Furthermore, clinically, maximum strength is more relevant than average strength. This helps to simplify and interpret the results in the sense that the aim is to explore whether the strength variations experienced after therapy are in the same direction and with the same intensity. In a second scenario, the same analysis was performed with respect to PROM variations. Finally, the relationships between the variations of the variables that underwent significant changes on both sides were analyzed in order to study whether these changes were similar.

For the statistical analysis of the last two hypotheses involving multiple comparisons, partial approximations have been made. A principal component analysis (PCA) was applied in order to reduce dimensionality and describe the dataset in terms of new uncorrelated variables that help to understand the main relationships between the study variables. Loadings allow us to interpret the distribution of our variables with respect to these principal components. A loading is large when its absolute value is >0.5. The sign will indicate whether the correlation between the variable and the component is positive or negative, resulting in a direct or inversely proportional relationship, respectively. As for the relationships of maximum strength and the relationships of the same variable comparing different sides, the comparisons were bivariate. Therefore, Pearson's or Spearman's correlation was applied depending on whether or not the normality criterion previously analyzed was met for the variables used to calculate the variation.

## Results

**Figure 2:**
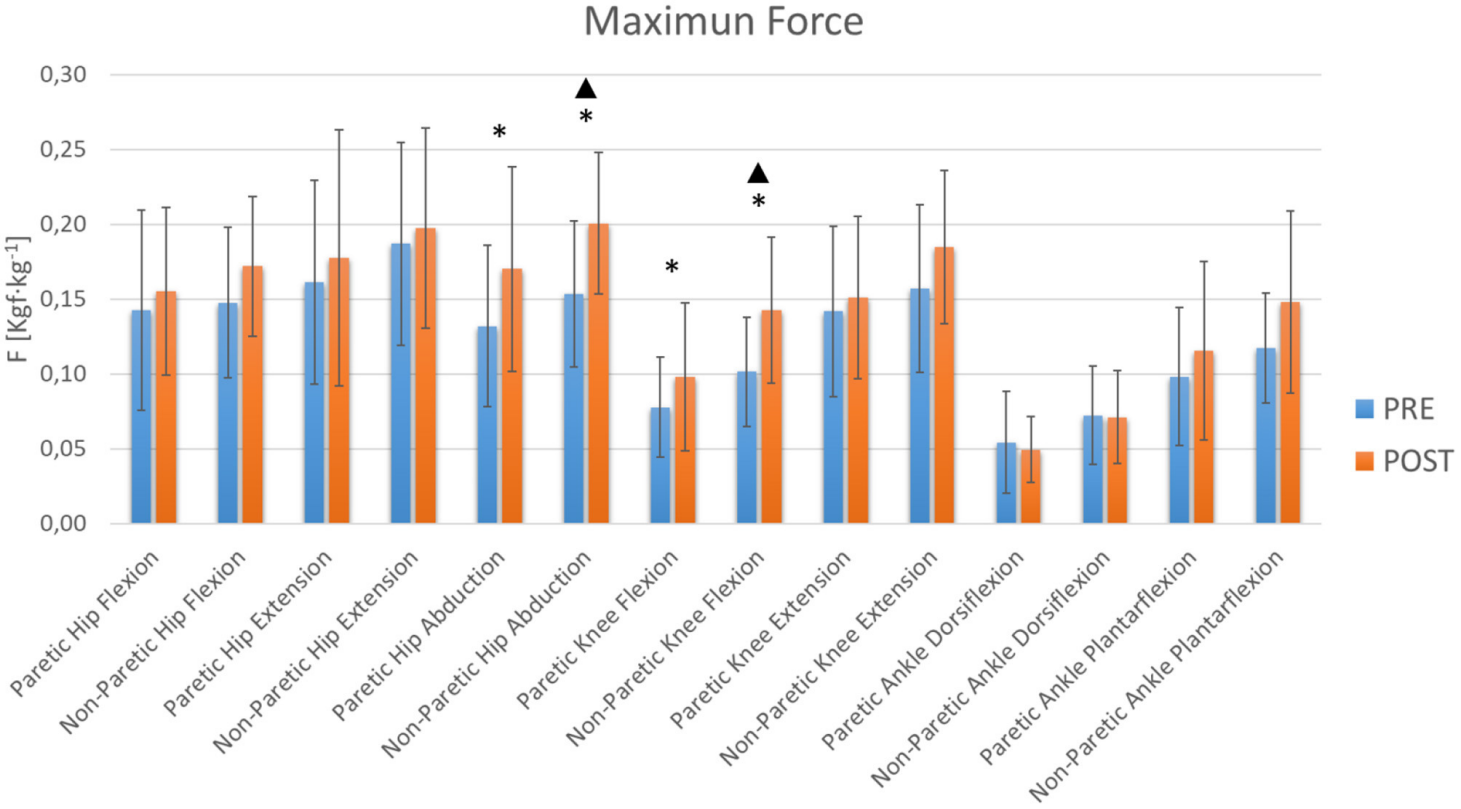
Maximum Lower Limb Forces. The blue and orange bars show the mean value (M). The standard deviation (SD) is also shown through error bars. *Significant differences. ^▴^Statistical power >80%.

**Figure 3:**
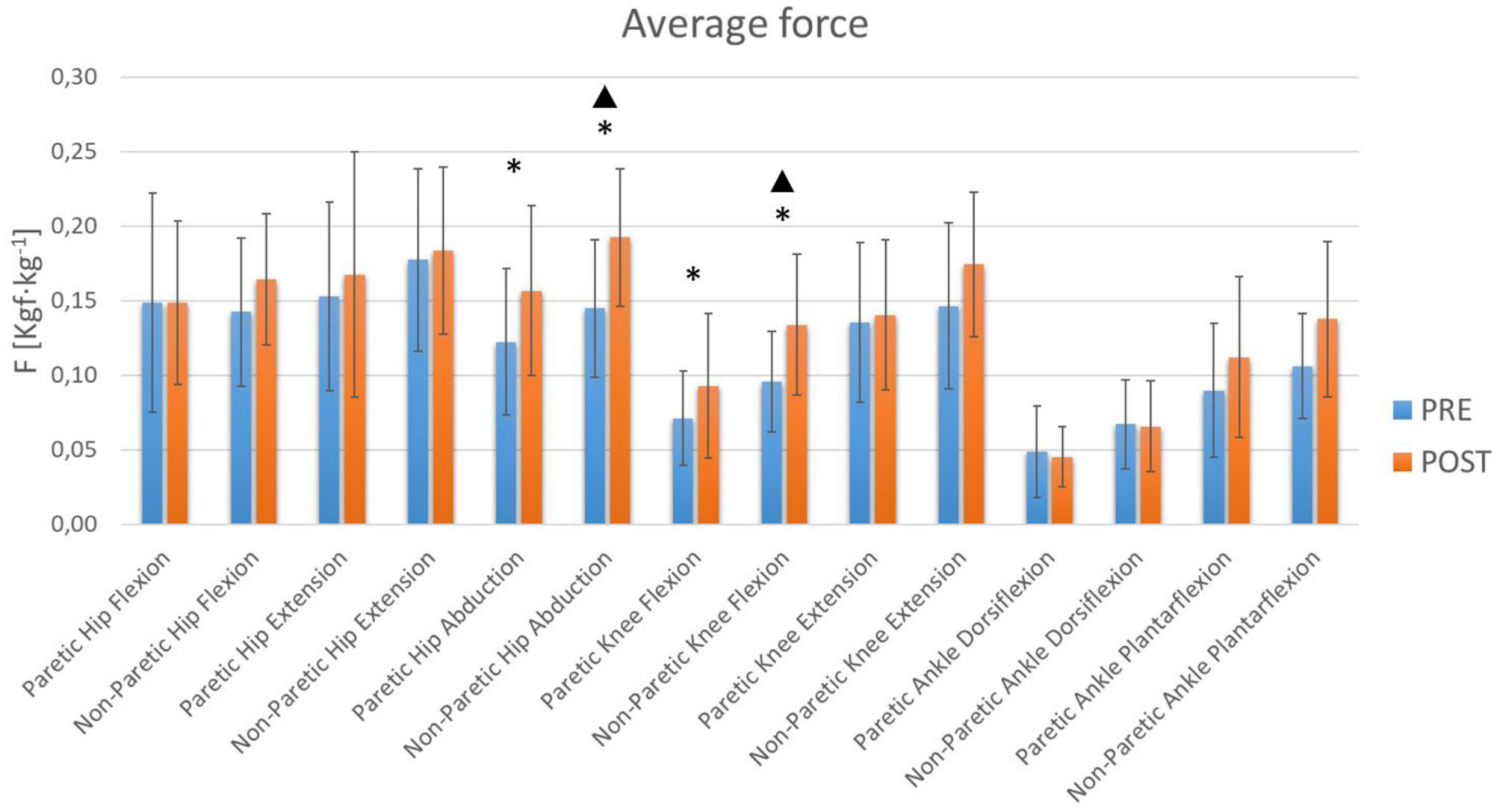
Average Lower Limb Forces. The blue and orange bars show the mean value (M). The standard deviation (SD) is also shown through error bars. *Significant differences. ^▴^Statistical power >80%.

From the results obtained in the hemiplegic patients (Table 1), certain statistically significant changes were observed (*p* < 0.05) which seem to indicate that this training therapy promotes strength gain mainly at the hip and knee joint level (Figures 2, 3). At the hip the maximum and average abduction strengths on the paretic side increased, although the statistical power was not sufficient (Table 1). However, the maximum and average abduction strengths of the non-paretic hip increased significantly with sufficient power (Table 1). At the knee, the maximum and average flexion strengths of the paretic side increased, but the observed power was low, while the maximum and average flexion strengths of the non-paretic side increased with adequate power (Table 1).

**Table 1:** Pre-therapy vs. post-therapy comparative statistical analysis.

**Assessment**	**Measurement**	***t*** **-Student or Wilcoxon**			***d***	**1-β**	**Pre**		**Post**	
		**Statistic**	***P*-value**	**α HB**			***M***	***SD***	***M***	***SD***
Dynamometry (kgf·kg^−1^)	Maximum paretic hip flexion	−1.155	0.260	0.005	0.24	0.52	0.14	0.07	0.16	0.06
	Average paretic hip flexion	−0.022	0.983	0.050	0.01	0.98	0.15	0.07	0.15	0.05
	Maximum non-paretic hip flexion	−2.305	0.031	0.003	0.48	0.51	0.15	0.05	0.17	0.05
	Average non-paretic hip flexion	−2.085	0.049	0.003	0.44	0.51	0.14	0.05	0.16	0.04
	Maximum paretic hip extension	−1.742	0.095	0.004	0.36	0.51	0.16	0.07	0.18	0.09
	Average paretic hip extension	−1.576	0.129	0.004	0.33	0.51	0.15	0.06	0.17	0.08
	Maximum non-paretic hip extension	−0.98	0.338	0.006	0.20	0.53	0.19	0.07	0.20	0.07
	Average non-paretic hip extension	−0.584	0.565	0.010	0.12	0.63	0.18	0.06	0.18	0.06
	**Maximum paretic hip abduction**	–**4.163**	**0.001**	**0.002**	**0.87**	**0.64**	**0.13**	**0.05**	**0.17**	**0.07**
	**Average paretic hip abduction**	–**4.316**	**0.001**	**0.002**	**0.90**	**0.69**	**0.12**	**0.05**	**0.16**	**0.06**
	**Maximum non-paretic hip abduction** ^▴^	–**5.789**	**0.001**	**0.002**	**1.21**	**0.96**	**0.15**	**0.05**	**0.20**	**0.05**
	**Average non-paretic hip abduction** ^▴^	–**5.837**	**0.001**	**0.002**	**1.22**	**0.97**	**0.14**	**0.05**	**0.19**	**0.05**
	**Maximum paretic knee flexion**	–**3.487**	**0.002**	**0.002**	**0.73**	**0.51**	**0.08**	**0.03**	**0.10**	**0.05**
	**Average paretic knee flexion**	–**3.541**	**0.002**	**0.002**	**0.74**	**0.53**	**0.07**	**0.03**	**0.09**	**0.05**
	**Maximum non-paretic knee flexion** ^▴^	–**4.856**	**0.001**	**0.002**	**1.01**	**0.83**	**0.10**	**0.04**	**0.14**	**0.05**
	**Average non-paretic knee flexion** ^▴^	–**4.865**	**0.001**	**0.002**	**1.01**	**0.83**	**0.10**	**0.03**	**0.13**	**0.05**
	Maximum paretic knee extension	−0.945	0.355	0.006	0.20	0.54	0.14	0.06	0.15	0.05
	Average paretic knee extension	−0.574	0.571	0.013	0.12	0.63	0.14	0.05	0.14	0.05
	Maximum non-paretic knee extension	−2.493	0.021	0.003	0.52	0.51	0.16	0.06	0.18	0.05
	Average non-paretic knee extension	−2.494	0.021	0.003	0.52	0.97	0.15	0.06	0.17	0.05
	Maximum paretic ankle dorsiflexion	0.711	0.484	0.007	0.15	0.58	0.05	0.03	0.05	0.02
	Average paretic ankle dorsiflexion	0.586	0.564	0.008	0.12	0.63	0.05	0.03	0.05	0.02
	Maximum non-paretic ankle dorsiflexion	−0.406^w^	0.685	0.017	0.04	0.69	0.07	0.03	0.07	0.03
	Average non-paretic ankle dorsiflexion	−0.336^w^	0.737	0.025	0.05	0.74	0.07	0.03	0.07	0.03
	Maximum paretic ankle plantarflexion	−1.576	0.129	0.005	0.33	0.51	0.10	0.05	0.12	0.06
	Average paretic ankle plantarflexion	−1.818	0.083	0.004	0.39	0.51	0.09	0.05	0.11	0.05
	Maximum non-paretic ankle plantarflexion	−1.802^w^	0.072	0.003	0.48	0.65	0.12	0.04	0.15	0.06
	Average non-paretic ankle plantarflexion	−2.256^w^	0.024	0.003	0.56	0.59	0.11	0.04	0.14	0.05
PROM (°)	Paretic hip flexion	−1.540^w^	0.124	0.007	0.14	0.20	118.87	8.35	117.70	11.23
	Nonparetic hip flexion	−0.935^w^	0.350	0.017	0.15	0.45	117.48	8.58	115.87	10.86
	Paretic hip extension	1.086	0.289	0.013	0.23	0.52	20.52	7.29	18.87	6.05
	Non-paretic hip extension	−2.045^w^	0.041	0.005	0.49	0.56	22.17	6.44	19.00	5.48
	**Paretic hip abduction** ^▴^	–**4.021**^**w**^	**0.001**	**0.002**	**1.45**	**1.00**	**38.17**	**6.63**	**26.52**	**7.63**
	**Non-paretic hip abduction** ^▴^	**5.504**	**0.001**	**0.002**	**1.15**	**0.94**	**40.09**	**9.79**	**27.48**	**7.54**
	**Paretic hip adduction**	–**3.247**^**w**^	**0.001**	**0.003**	**0.82**	**0.53**	**25.22**	**6.17**	**19.43**	**5.30**
	**Non-paretic hip adduction** ^▴^	**6.049**	**0.001**	**0.002**	**1.26**	**0.98**	**26.61**	**4.76**	**18.39**	**3.86**
	Paretic hip external rotation	−0.767^w^	0.443	0.025	0.19	0.60	29.26	6.34	27.61	5.69
	Non-paretic hip external rotation	2.471	0.022	0.005	0.52	0.51	31.30	6.57	27.91	5.23
	Paretic hip internal rotation	3.213	0.004	0.003	0.67	0.51	29.74	6.44	25.04	8.01
	Non-paretic hip internal rotation	3.195	0.004	0.003	0.667	0.51	33.48	6.72	28.43	5.87
	Paretic knee flexion	1.79	0.087	0.006	0.37	0.51	125.96	10.39	122.48	10.87
	Non-paretic knee flexion	−1.779^w^	0.075	0.006	0.39	0.49	126.35	8.50	122.83	9.45
	Paretic knee extension	−0.254^w^	0.799	0.050	0.02	0.80	2.96	6.63	2.87	4.17
	Non-paretic knee extension	−1.265^w^	0.206	0.010	0.29	0.54	1.22	5.11	2.35	3.79
	Paretic ankle dorsiflexion	−2.861^w^	0.004	0.003	0.65	0.45	8.78	6.79	5.57	6.22
	Non-paretic ankle dorsiflexion	−2.361^w^	0.018	0.004	0.53	0.48	12.04	6.50	9.17	6.84
	**Paretic ankle plantarflexion**	**4.508**	**0.001**	**0.002**	**0.94**	**0.75**	**42.61**	**9.66**	**34.57**	**8.90**
	**Non-paretic ankle plantarflexion** ^▴^	–**3.642**^**w**^	**0.001**	**0.003**	**1.20**	**0.95**	**41.74**	**7.32**	**31.96**	**8.21**
	Paretic ankle inversion	−1.509^w^	0.131	0.008	0.38	0.58	20.09	5.13	18.43	4.97
	Non-paretic ankle inversion	−2.315^w^	0.021	0.004	0.54	0.53	21.48	4.52	18.91	5.24
	Paretic ankle eversion	−2.800^w^	0.005	0.004	0.70	0.56	4.96	3.99	2.61	2.23
	Non-paretic ankle eversion	3.219	0.004	0.003	0.67	0.52	6.70	3.50	4.35	3.31

*Wilcoxon tests are marked with a letter “w”, the rest complied with normality and t-Student could be applied. Significant results obtained after Holm-Bonferroni (HB) adjustment are highlighted in bold*.

▴ *Significant changes with statistical power >80%*.

*Mean (M) and standard deviation (SD) were included*.

**Figure 4:**
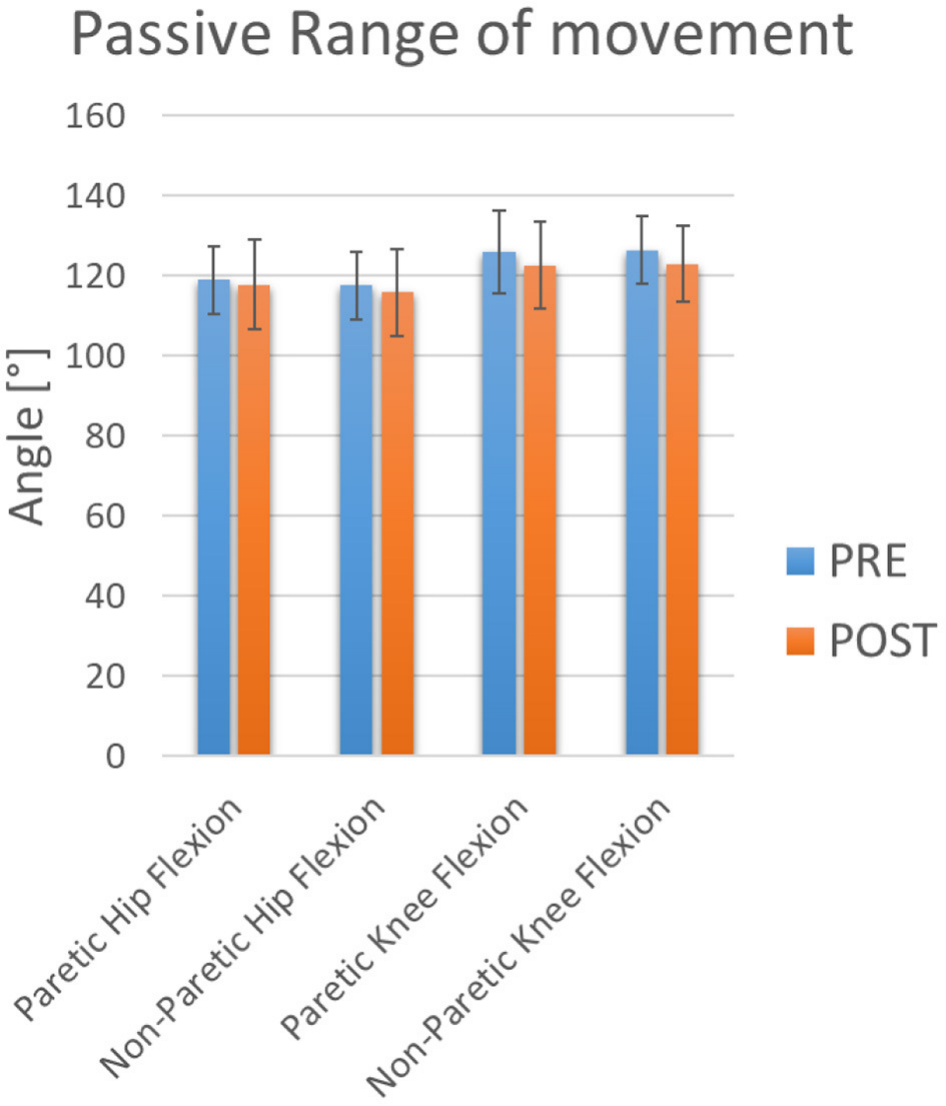
PROM Flexion. The blue and orange bars show the mean value (M). The standard deviation (SD) is also shown through error bars.

**Figure 5:**
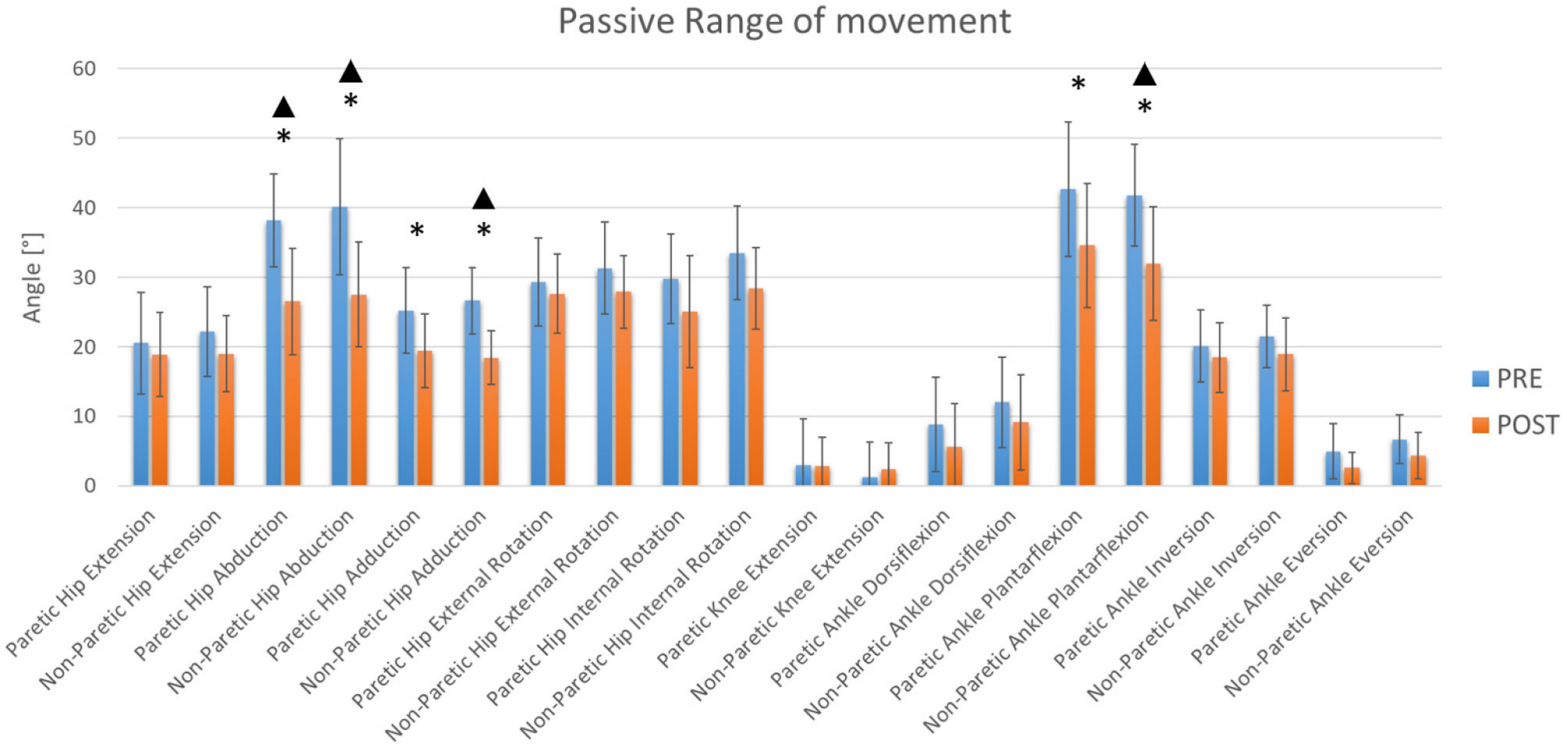
Lower limbs PROM. The blue and orange bars show the mean value (M). The standard deviation (SD) is also shown through error bars. *Significant differences. ^▴^Statistical power >80%.

Training also seems to generate changes in PROM at the hip and ankle (Figures 4, 5). However, in relation to PROM, significant decreases were observed in hips and ankles. A decrease in adduction on the paretic side was observed. However, not enough statistical power was obtained (Table 1). At the hip joint level, a decrease in abduction on both sides and adduction on the non-paretic side was also observed with sufficient statistical power (Table 1). At the ankle, a significant decrease was observed on both sides for plantarflexion (Table 1). However, sufficient statistical power was only obtained for plantarflexion of the non-paretic ankle (Table 1). It was also observed that there was no significant change in the average hip flexion strength on the paretic side, in the average non-paretic knee extension or in the extension PROM of the paretic knee. These results show sufficient power (Table 1).

As detailed above, the percentage of variation was calculated for those variables that showed significant changes (Table 2). For these variables a bivariate correlation (Table 3) or PCA (Table 4) analysis was applied, as detailed in Section Statistical Analysis. Table 4 with the results of the PCA of Paretic PROM shows the relevant principal components that explain most of the variance and loadings. With regard to scenario 1 detailed in Section Statistical Analysis, a Pearson's test was applied to obtain the relationship between the variations of the maximum strengths that showed significant changes. However, the correlation between the variation of maximum abduction of the hip and the variation of maximum flexion of the knee on each side were not significant (Table 3). With regard to scenario 2, PCA was applied for both sides. On the paretic side, the components were found to explain 76% of the variance (Table 4). Strong positive correlation of hip adduction PROM variation and hip abduction PROM variation is observed for component 1 and a positive correlation of ankle plantarflexion PROM variation for component 2. As for the non-paretic side, the principal components explained 62% of the variance. Only one component was extracted and there were no significant correlations. Additionally, Table 3 shows a large and significant positive correlation between the maximum variation of paretic hip abduction strength and the maximum variation of non-paretic hip abduction strength. Positive mid-level correlations were also observed between the variation of the paretic hip adduction PROM and the variation of the non-paretic hip adduction PROM and between the variation of the paretic ankle plantarflexion PROM and the variation of the non-paretic ankle plantarflexion PROM.

**Table 2:** Changes in dynamometry and PROM.

**Assessment**	**Variable of interest**	***M* (%)**	***SD***
Dynamometry	Maximum paretic hip abduction variation	33.15	42.38
	Average paretic hip abduction variation	33.29	42.62
	Maximum non-paretic hip abduction variation	37.71	40.68
	Average non-paretic hip abduction variation	40.00	41.86
	Maximum paretic knee flexion variation	24.59	38.17
	Average paretic knee flexion variation	29.54	43.86
	Maximum non-paretic knee flexion variation	54.85	71.33
	Average non-paretic knee flexion variation	50.71	63.56
PROM	Paretic hip abduction variation	−29.96	18.46
	Non-paretic hip abduction variation	−28.39	22.84
	Paretic hip adduction variation	−20.11	24.42
	Non-paretic hip adduction variation	−28.38	21.16
	Paretic ankle plantarflexion variation	−17.25	19.01
	Non-paretic ankle plantarflexion variation	−22.50	18.93

*Mean (M) and standard deviation (SD) were included*.

**Table 3:** Correlations between average percentages of variation.

**Assessment**	**Variable of interest 1**	**Variation of variable of interest 2**	**Correlation coefficient**	**Sig**.	***d***	**1-β**
Dynamometry	Maximum paretic hip abduction variation	Maximum paretic knee flexion variation	0.355 (P)	0.097	0.60	0.93
	Maximum non-paretic hip abduction variation	Maximum non-paretic knee flexion variation	0.242 (P)	0.266	0.49	0.91
	**Maximum paretic hip abduction variation**	**Maximum non-paretic hip abduction variation**	**0.728 (P)**	0.001^**^	**0.85**	**0.99**
	Maximum paretic knee flexion variation	Maximum non-paretic knee flexion variation	0.147 (P)	0.504	0.38	0.89
PROM	Paretic hip abduction variation	Non-paretic hip abduction variation	0.367 (S)	0.085	0.61	0.93
	**Paretic hip adduction variation**	**Non-paretic hip adduction variation**	**0.490 (S)**	0.018^*^	**0.70**	**0.94**
	**Paretic ankle plantarflexion variation**	**Non-paretic ankle plantarflexion variation**	**0.522 (S)**	0.011^*^	**0.72**	**0.95**

** *Correlation is significant at the 0.01 level (bilateral)*.

* *Correlation is significant at the 0.05 level (bilateral). Pearson (P) and Spearman (S)*.

*Significant results are highlighted in bold*.

**Table 4:** PCA Paretic PROM (76% explained variance).

**Rotated component matrix^a^**		
**Variable of interest**	**Component**	
	**1**	**2**
Hip adduction PROM variation	**0.827**	−0.182
Hip abduction PROM variation	**0.753**	0.285
Ankle plantarflexion PROM variation		**0.961**

a *Rotation converged in 3 iterations*.

*Extraction Method: Principal Component Analysis. Rotation Method: Varimax with Kaiser Normalization*.

*Relevant changes in bold*.

Finally, Supplementary Table S1 presents the torque of the exoskeleton joints extracted from the LabVIEW interface database. The average maximum torque during step with load exercise is presented for each joint considering torque sensor values at baseline and post-intervention condition to describe the interaction of the device with the user. Supplementary Figures S2–S6 show an example of recordings that were made during the trainings with one patient during the robotic therapy.

## Discussion

This study was conducted over 12 weeks to investigate the effectiveness of this new approach combining an exoskeleton-based robotic platform with the Bobath concept for the therapeutic treatment of the lower limbs in stroke patients. The therapist who carried out the therapy with the users reports that, throughout the sessions, the patients synchronized more easily with the exoskeleton, coordinating the start of the exercise with the programed sound and accompanying the movement of the exoskeleton with active-assisted movement. This implies that the patient learns the movement pattern as a consequence of continuous repetition, which generated afferent information to the central nervous system, mainly from the joint and musculoskeletal receptors. Similarly, patients report less difficulty in executing the movement throughout the sessions. Therefore, the usefulness of this new platform for transferring exercises performed in conventional therapies to robot-assisted therapies has been demonstrated. This platform simplifies the training task by reducing the workload of physiotherapists, who will be in charge of supervising and configuring the training. In this way, rehabilitation exercises, previously recorded in the motion capture system, can be guided by robotic assistance for their correct execution in the therapeutic treatments.

Studies have shown that lower limbs muscle strength has a moderate relationship with functional gait capacity and gait speed (Pennycott et al., 2012; Menezes et al., 2020). The results show that with this therapy there is a significant increase on the non-paretic side in hip abduction strength and knee flexion strength. Other significant increases in strength were also observed, despite not obtaining sufficient statistical power, in paretic hip abduction and paretic knee flexion. It is curious that although the exoskeleton allows movement in the sagittal plane, it appears that the hip is still able to exercise control of movement in the frontal plane. In addition, a general trend of increased muscle strength was observed in all joints, although no other significant changes were achieved. This suggests that this type of robotic therapy based on the Bobath method is useful for improving muscle strength in the lower extremities, with the greatest effect at the hip and knee joints.

These results are consistent with other similar studies, such as the work of Kayabinar et al. (2019) which compared the effects of robotic and conventional gait training based on the Bobath method. This study demonstrated the effectiveness of robotic and conventional therapy for the rehabilitation of stroke patients in terms of mobility, quality of life and balance; furthermore, its application in the clinical setting is reliable (Kayabinar et al., 2019). Other studies, such as the randomized controlled trial by Kim et al. (2019), reported benefits associated with electromechanical assisted gait training with Morning Walk, showing improvements in lower leg muscle strength and balance in patients with hemiparesis compared to the control group (Kim et al., 2018). The main advantage of our study is that we had control of each of the lower limb joints involved in walking, and we could vary the assistance in each of them independently, adapting to the patient's needs. The use of the Lokomat allowed the mobilization of hips and knees, whereas the Morning Walk only allowed good control of the movement of the ankle (Kim et al., 2018).

Prevention of secondary impairment and promotion of a state of functional independence aim to reduce spasticity and increase range of motion (Wu et al., 2011; Kim et al., 2014). However, in our study, negative changes were observed at the hip and ankle levels. At the hip, there was a decrease in the frontal plane in abduction and adduction movement. This may be related to the performance of exercises that focus primarily on movement in the sagittal plane, where the H3 exoskeleton had movement capacity (Technaid, 2020). At the ankle joint level, the PROM was observed in the sagittal plane bilaterally for plantarflexion movements. In addition, a tendency to decrease the PROM is observed, which leads us to think that it is necessary to combine this therapy with stretching exercises to avoid these negative effects.

Regarding the statistical study on the relative rates of change of the variables that underwent significant changes, the maximum hip abduction strength on both sides showed a strong positive correlation (Table 2). This seems to indicate that the increase in hip abduction strength on both sides was balanced. However, at knee level, no similar changes were experienced. This seems to indicate that the non-paretic side continued to compensate for the paretic side, so it would be interesting to find a more effective method of adjusting assistance to achieve a balanced final strengthening on both sides. Furthermore, this also seems to indicate that the exercises proposed in the therapy may require a greater effort of the hip and knee joints, where most of the significant changes found were observed. The PCA results for PROM variations for the paretic side showed a high correlation between hip abduction and adduction movements. However, the PCA results for PROM variations explained the variance to a lesser extent for non-paretic side. This is because the correlations were low overall. This seems to indicate that the PROM reduction effects observed after therapy were generalized, but the evolution was different for each joint movement.

A concept that may help to understand the relevance of these results is the minimal clinically important difference (MCID). MCID is defined as “the smallest difference in score in the domain of interest which patients perceive as beneficial and which would mandate, in the absence of troublesome side effects and excessive cost, a change in the patient's management” (Jaeschke et al., 1989). This concept is common in the clinic and some studies have analyzed it in post-stroke gait for different joints in the sagittal plane such as the hip, where the MCID of the ROM for the affected side is about 5.81° and for the unaffected side at around 2.86° (Guzik et al., 2021), and the knee, where MCID of the ROM for the affected side is about 8.48° and for the unaffected side at around 6.81° (Guzik et al., 2020). Although PROM is related to the ROM studied in Guzik et al. (2020, 2021), it is usually higher and no specific MCID was found for this population and measured in the literature. Neither was it found for lower limb joint strength. Nevertheless, obtaining statistical significance and power helped to identify those variables that showed a relevant change. Even so, it is considered of special interest to obtain in future studies a representative MCID for this population in relation to the study variables, in order to facilitate the interpretation of the therapy outcomes. Overall, it can be useful to adjust the robot-aided training according this type of objective assessment of the patient's performance during the course of therapy.

The findings suggest that more exercises need to be incorporated into therapy where greater involvement of the ankle joint is required, such as the standing calf, to achieve significant benefits in this joint. In addition, since the spasticity of a paralyzed muscle is closely related to the speed of movement of the joint, it is necessary to include exercises for the maintenance and improvement of range of motion, such as passive stretching exercises, to maintain muscle flexibility by decreasing joint stiffness. This may facilitate the generation of muscle strength (Wu et al., 2011; Pennycott et al., 2012; Dae-Yeon and Wan-Young, 2020).

Similar results were obtained among the different volunteers who participated in the experiment, but there was a great variation in the results between them, which leads us to think that it is necessary to adapt the percentage of assistance to each specific case in each of the joints during training. To this end, it is necessary to establish an objective action protocol that determines to what specific degree the assistance provided by the technology should be reduced over the course of the sessions, depending on the patient's evolution.

Although positive changes in strength were obtained, it is a therapy that targets a specific impairment, which may produce limited effects and therefore often does not lead to improvements in function (Pennycott et al., 2012). However, it would be necessary to study the effects at the biomechanical level and with other clinical trials to clarify the extent of the benefits of therapy. One limitation that has been found is that the exoskeleton works in the sagittal plane and therefore limits other movements. Because most exoskeletons tend to act only in the sagittal plane, the lack of actuation in other planes of motion, such as the frontal plane, reduces the capability of these devices to increase or maintain lateral stability and provide active lateral weight shifting. If the robot assists lateral movement, it could increase the gait stability and also reduce the use of external balance aids such as walkers and crutches, allowing patients to walk with the exoskeleton hands-free (Wang et al., 2013). On the other hand, it would be necessary to increase the sample size to identify other changes of interest that did not obtain sufficient statistical power. For future work, it is necessary to combine this methodology with passive stretching exercises and to include functional exercises that may generate better and more positive results.

## Conclusions

The usefulness of this new platform for transferring exercises performed in conventional therapies to robot-assisted therapies for gait rehabilitation has been demonstrated. Moreover, it has been proven that the application of certain exercises based on the Bobath concept can be useful to increase muscle strength. This parameter is related to functional gait ability and gait speed and is affected after stroke. However, these exercises should be complemented by other therapeutic exercises focused on gait rehabilitation, such as exercises for maintaining and increasing of range of motion that help to reduce spasticity and strength recovery. In addition, it is necessary to establish an objective protocol detailing the criteria for the choice of the amount of assistance required at any given time during therapy for each joint according to the patient's needs. Therefore, an appropriate combination of exercises in robotic-assisted therapy and an objective criterion for the selection of the percentage of assistance based on the patient's needs can help improve gait rehabilitation treatment.

## Data Availability Statement

The raw data supporting the conclusions of this article will be made available by the authors, without undue reservation.

## Ethics Statement

The studies involving human participants were reviewed and approved by Local Ethical Committee of the Rehabilitation Center Club de Leones Cruz del Sur. The patients/participants provided their written informed consent to participate in this study.

## Author Contributions

JM revised the entire article. JG-C and JM conceived and structured the contents of the paper. JG-C conducted the article search, revised the integration of the different sections, performed the data processing, statistical analysis, and wrote the draft of the manuscript. PB designed and developed the protocols, the experimentation, recruited the participants, carried out the follow-up, training and tests on the participants, reviewed, and edited the article. RC and AM programmed the software used in the study. JM, KA, AG, and JA revised the entire article. All the authors read and approved the final manuscript.

## Funding

This study was sponsored and financially supported by Innovation and Competitiveness Fund 2017 of the Regional Government of Magallanes and Chilean Antarctica, Punta Arenas, Chile (BIP code Number 30488844-0) and by the Rehabilitation Center “Club de Leones Cruz del Sur”, Punta Arenas, Chile. This work has been also partially funded by CSIC Interdisciplinary Thematic Platform (PTI+) NEURO-AGING+ (PTI-NEURO-AGING+).

## Conflict of Interest

The authors declare that the research was conducted in the absence of any commercial or financial relationships that could be construed as a potential conflict of interest.

## Publisher's Note

All claims expressed in this article are solely those of the authors and do not necessarily represent those of their affiliated organizations, or those of the publisher, the editors and the reviewers. Any product that may be evaluated in this article, or claim that may be made by its manufacturer, is not guaranteed or endorsed by the publisher.
