# Supplementary material for: A Robot-Assisted Therapy to Increase Muscle Strength in Hemiplegic Gait Rehabilitation
Source: Front Neurorobot. 2022 Apr 29;16:837494. doi: 10.3389/fnbot.2022.837494 (PMC9100587; doi:10.3389/fnbot.2022.837494)

Supplementary Material

Table S1 – Torque sensor values from exoskeleton actuators during step with load exercise

Joint exoskeleton joints	Condition	
	Baseline	Post Intervention
**Right hip Sensor Torque (Nm)**	24,76	22,88
**Right knee Sensor Torque (Nm)**	4,07	4,88
**Right ankle Sensor Torque (Nm)**	22,16	14,7
**Left hip Sensor Torque (Nm)**	26,37	17,94
**Left knee Sensor Torque (Nm)**	28,03	22,24
**Left ankle Sensor Torque (Nm)**	23,82	21,44

The table presents the average torque data recorded during the step support exercise sessions with load on the left lower extremity.

Figure S1 – Control diagram

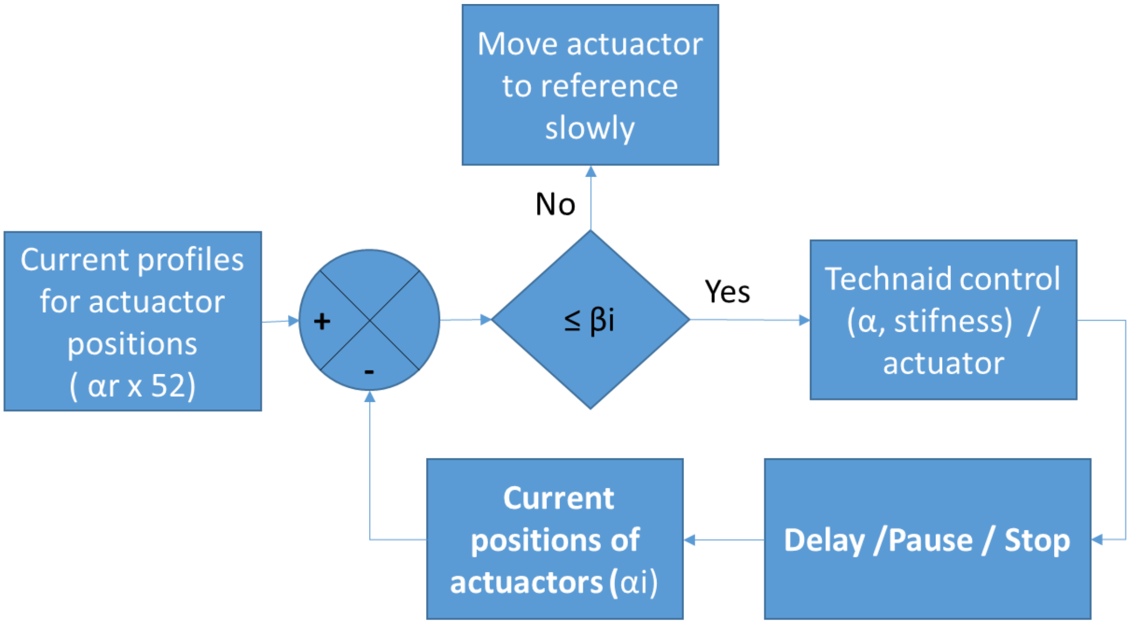

Figures S2 to S6 show an example of the recordings that were made for one patient during therapy. Each figure shows one cycle of the proposed exercise. These figures are composed of 6 graphs corresponding to the cycle executed by each joint. Each cycle has been divided into 51 points, which is the standard sampling per movement cycle from the VICON supplier. The movement cycle is then normalized to percentage of movement.

Figure S2 - Interface assistance configuration

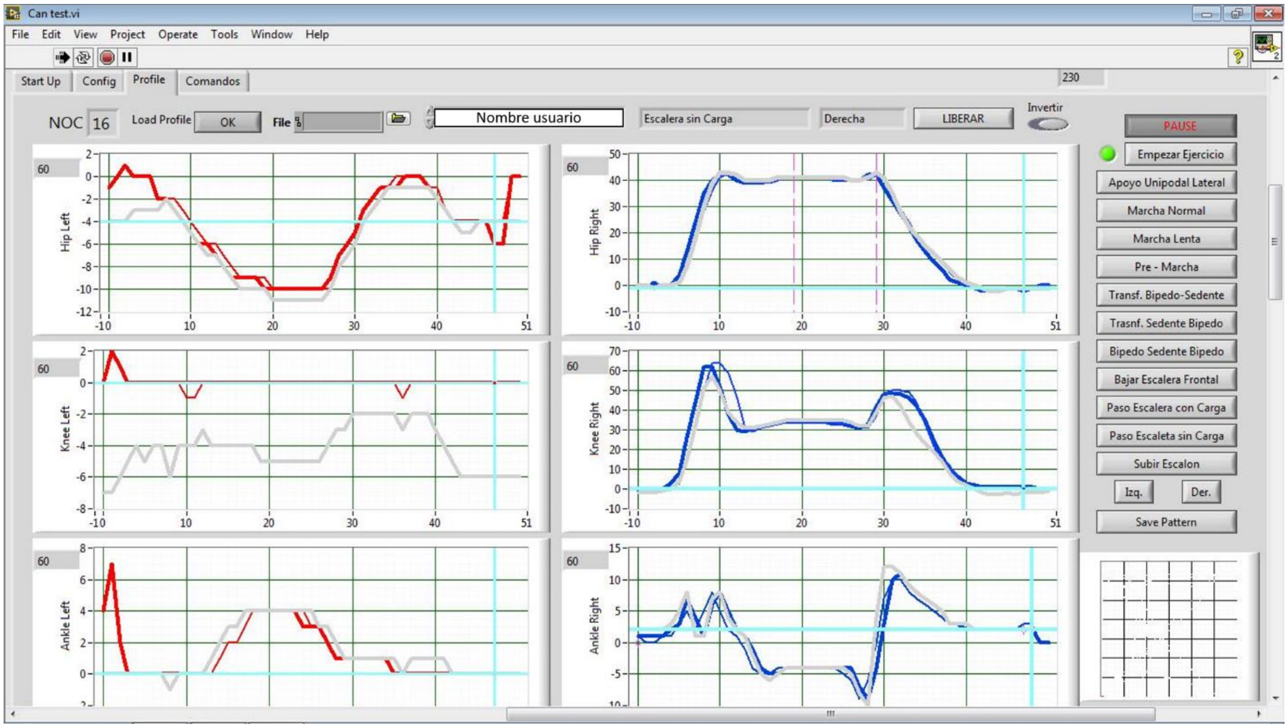

Figure S2 shows a cycle for each joint with the kinematic curve programmed (Profile) and the kinematics executed ("joint" kinematics) by the user. The assistance is configured in percentage in each of the grey boxes of each graph. In this case, an assistance of 60% is configured for all joints.

Figures S3 to S6 show the experimental recordings where the programmed kinematics (Profile), the executed kinematics (“joint” Kinematics), the motor torque and the torque recorded (Sensor Torque) by the sensors for all the joints in both lower limbs are presented. Each graph has four curves. The grey line corresponds to the kinematic trajectory programmed into the exoskeleton measured in degrees (reference motion). The light blue line represents the kinematic trajectory executed by the patient during the exercise measured in degrees (actual movement). The orange line is the torque measured on the exoskeleton levers in Nm (integration between the user and the robotic assistance). The blue line represents the torque measured on the motors during exercise (robotic assistance).

Figure S3 – Right step exercise with load

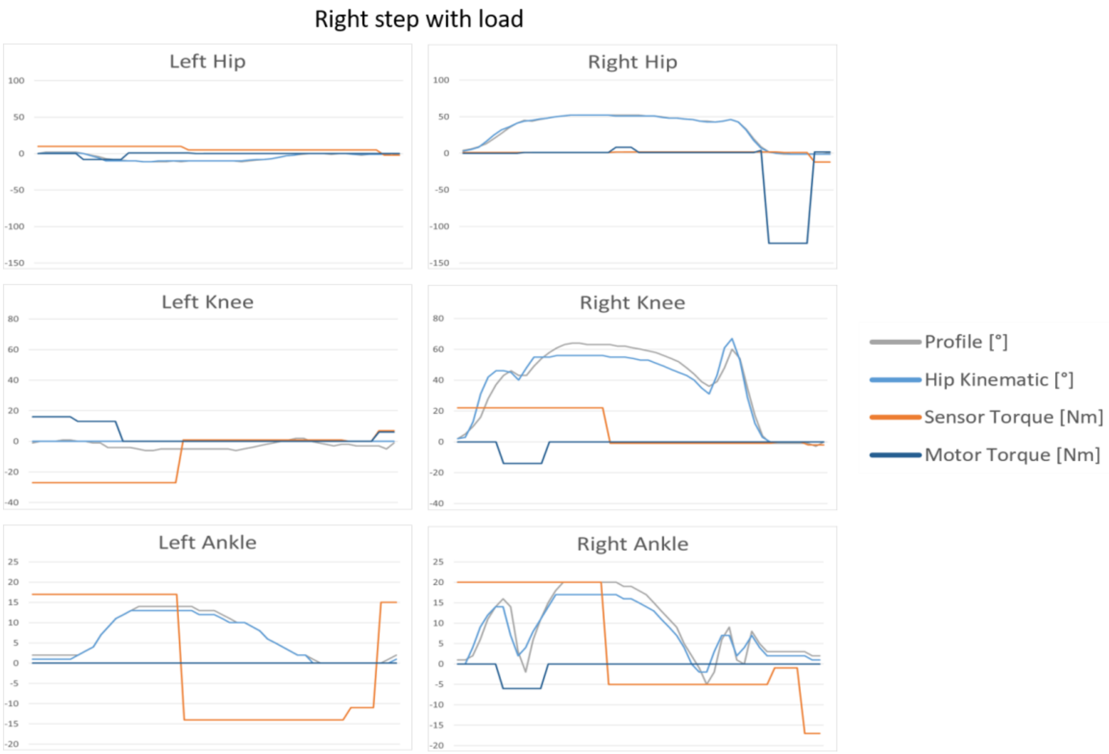

Figure S4 - Right step exercise without load

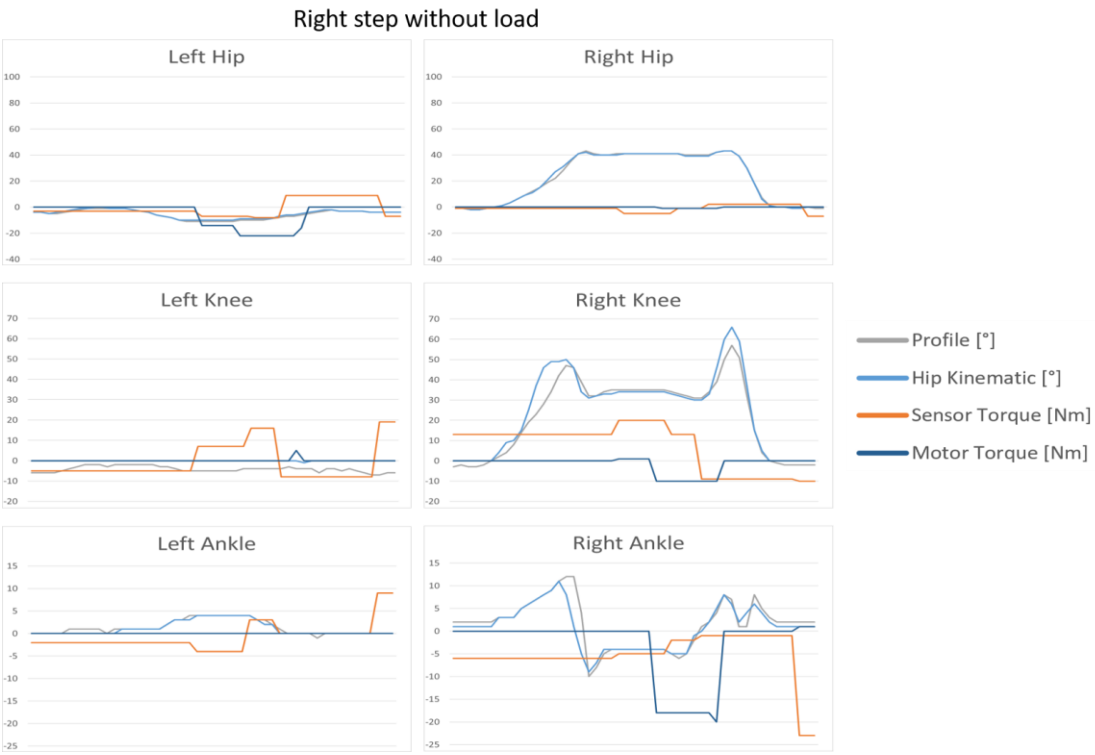

Figure S5 - Right pre-gait exercise

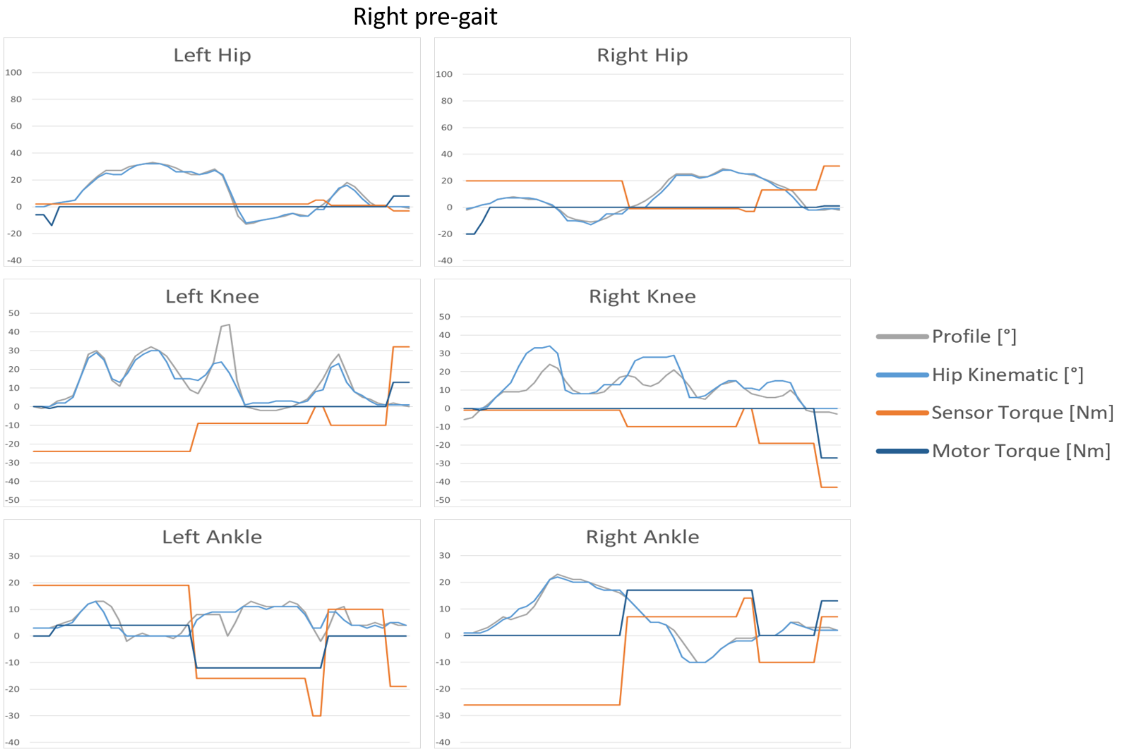

Figure S6 – Sit-to-stand transfer exercise

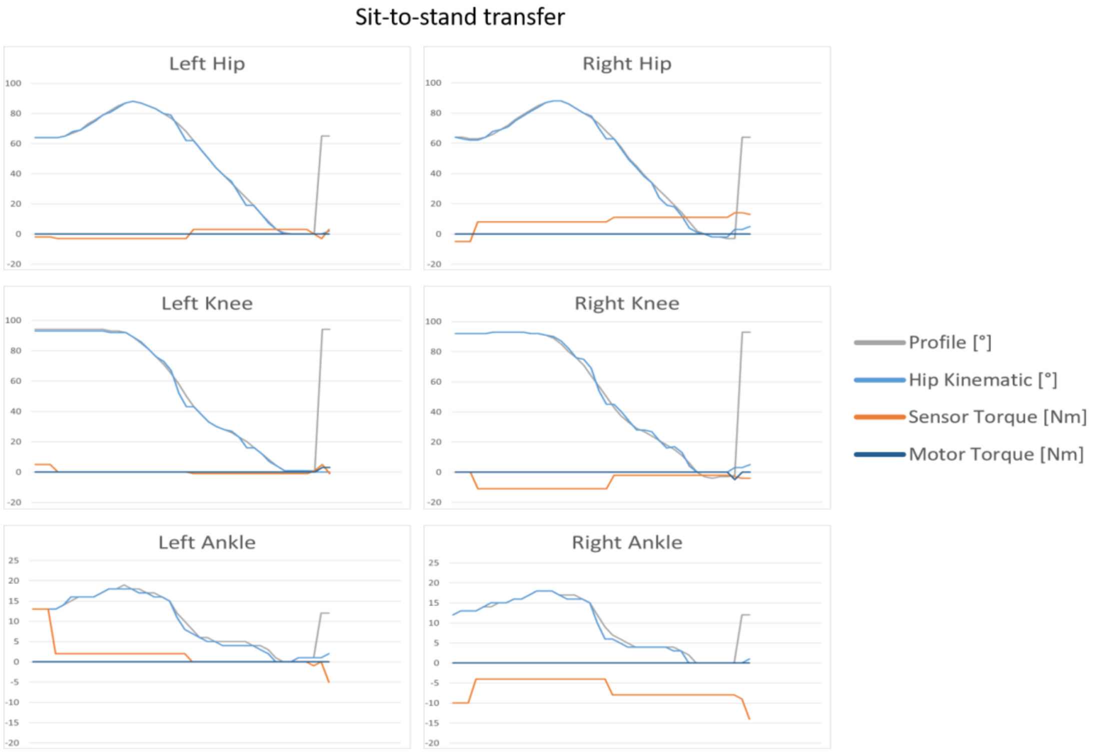

Supplement: Supplementary file 1 [file Data_Sheet_1.pdf]
